# Analytical Characterization of 3-MeO-PCP and 3-MMC in Seized Products and Biosamples: The Role of LC-HRAM-Orbitrap-MS and Solid Deposition GC-FTIR

**DOI:** 10.3389/fchem.2020.618339

**Published:** 2021-02-08

**Authors:** Giampietro Frison, Flavio Zancanaro, Samuela Frasson, Laura Quadretti, Michele Agnati, Francesca Vlassich, Giuseppe Gagliardi, Tania Maria Grazia Salerno, Paola Donato, Luigi Mondello

**Affiliations:** ^1^ Laboratory of Environmental Hygiene and Forensic Toxicology, DMPO Department, AULSS 3, Venice, Italy; ^2^ Emergency Department Unit, Madonna della Salute Hospital, AULSS 5, Porto Viro (Rovigo), Italy; ^3^ Intensive Care Unit, Madonna della Salute Hospital, AULSS 5, Porto Viro (Rovigo), Italy; ^4^ Department of Anesthesiology and Intensive Care, AULSS 5, Porto Viro (Rovigo), Italy; ^5^ BeSep S.r.l., c/o Department of Chemical, Biological, Pharmaceutical and Environmental Sciences, University of Messina, Messina, Italy; ^6^ Department of Biomedical, Dental, Morphological and Functional Imaging Sciences, University of Messina, Messina, Italy; ^7^ Department of Chemical, Biological, Pharmaceutical and Environmental Sciences, University of Messina, Messina, Italy; ^8^ Chromaleont S.r.l., c/o Department of Chemical, Biological, Pharmaceutical and Environmental Sciences, University of Messina, Messina, Italy; ^9^ Research Unit of Food Science and Nutrition, Department of Science and Technology for Humans and the Environment, Campus Bio-Medico University of Rome, Rome, Italy

**Keywords:** 3-methoxyphencyclidine, 3-methylmethcathinone, non-fatal intoxication, forensic toxicology, liquid chromatography-high-resolution mass spectrometry, gas chromatography, fourier transform infrared spectroscopy, solid deposition GC-FTIR interface

## Abstract

Among the phencyclidine (PCP) and synthetic cathinone analogs present on the street market, 3-methoxyphencyclidine (3-MeO-PCP) is one of the most popular dissociative hallucinogen drugs, while 3-methylmethcathinone (3-MMC) is a commonly encountered psychostimulant. Numerous 3-MeO-PCP- and 3-MMC-related intoxication cases have been reported worldwide. Identification of the positional isomers of MeO-PCP and MMC families are particularly challenging for clinical and forensic laboratories; this is mostly due to their difficult chromatographic separation (particularly when using liquid chromatography–LC) and similar mass spectrometric behaviors. 3-MeO-PCP and 3-MMC were identified in two powders, detained by two subjects and seized by the police, by different analytical techniques, including liquid chromatography-high-resolution accurate-mass Orbitrap mass spectrometry (LC-HRAM-Orbitrap-MS), and solid deposition gas chromatography-Fourier transform infrared spectroscopy (sd-GC-FTIR). LC-HRAM-Orbitrap-MS allowed us to assign the elemental formulae C_18_H_27_NO (MeO-PCP) and C_11_H_15_NO (MMC) through accurate mass measurement of the two MH^+^ ions, and the comparison of experimental and calculated MH^+^ isotopic patterns. However, MH^+^ collision-induced product ions spectra were not conclusive in discriminating between the positional isomers [(3-MeO-PCP vs. 4-MeO-PCP) and (3-MMC vs. 4-MMC and 2-MMC)]. Likewise, sd-GC-FTIR easily allowed us to differentiate between the MeO-PCP and MMC positional isomers unambiguously, confirming the presence of 3-MeO-PCP and 3-MMC, due to the high-quality match factor of the experimental FTIR spectra against the target FTIR spectra of MeO-PCP and MMC isomers in a dedicated library. 3-MeO-PCP (in contrast to 3-MMC) was also detected in blood and urine samples of both subjects and analyzed in the context of routine forensic casework by LC-HRAM-Orbitrap-MS following a simple deproteinization step. In addition, this untargeted approach allowed us to detect dozens of phase I and phase II 3-MeO-PCP metabolites in all biological specimens. Analysis of the extracted samples by sd-GC-FTIR revealed the presence of 3-MeO-PCP, thus confirming the intake of such specific methoxy-PCP isomer in both cases. These results highlight the effectiveness of LC-HRAM-Orbitrap-MS and sd-GC-FTIR data in attaining full structural characterization of the psychoactive drugs, even in absence of reference standards, in both non-biological and biological specimens.

## Introduction

The new psychoactive substances (NPS) phenomenon, characterized by peculiar features as opposed to the traditional drug phenomena (Peacock et al., 2019; Zamengo et al., 2019), has emerged as a global threat that challenges public health and institutions (UNODC, 2020). Psychoactive and physical effects, toxicity, addiction potential, and potency may vary dramatically among specific NPS. Furthermore, individual products may contain multiple psychoactive ingredients, adulterants, or by-products in extremely variable concentrations, exposing consumers to unpredictable doses of multiple NPS with serious health-related consequences, especially in young people (Zamengo et al., 2014; Orsolini et al., 2019; Larabi et al., 2020). According to their effects, dissociatives, sedatives/hypnotics, synthetic opioids, hallucinogens, synthetic cannabinoid receptor agonists, and psychostimulants are the NPS that most frequently appear on the recreational drug market (UNODC, 2020).

Synthetic cathinones include a very large number of substances with psychostimulant effects, elicited by augmenting central monoamine transmission through release facilitation and/or presynaptic transport inhibition (Simmons et al., 2018). 3-methylmethcathinone (3-MMC) is a commonly encountered synthetic cathinone, introduced in the NPS market to replace mephedrone (4-methylmethcathinone, 4-MMC), and it has seen rapid spread among drug users (Ferreira et al., 2019; Larabi et al., 2019a). 2-methylmethcathinone (2-MMC) has also been reported, albeit more rarely (Nycz et al., 2016). Several 3-MMC-related non-fatal and fatal intoxication cases have been described (Bäckberg et al., 2015b; Jamey et al., 2016; Ameline et al., 2019a; Margasińska-Olejak et al., 2019).

Phencyclidine (PCP)-based compounds are a group of dissociative hallucinogen NPS that exert their primary pharmacologic effect through blockade of excitatory N-methyl-D-aspartate (NMDA) receptors. Additional effects, as relatively weak opioid and dopamine receptor agonists and on serotonergic and noradrenergic pathways, have been described (Morris and Wallach, 2014; Wallach et al., 2014; Wallach and Brandt, 2018). Among the PCP analogs present on the street market, 3-methoxyphencyclidine (3-MeO-PCP) is one of the most popular ones. Even 4-methoxyphencyclidine (4-MeO-PCP) and 2-methoxyphencyclidine (2-MeO-PCP) have been synthesized (Maddox et al., 1965). 4-MeO-PCP is less potent than 3-MeO-PCP, while 2-MeO-PCP is very weak (Kalir, 1981; Wallach and Brandt, 2018), probably explaining why the latter is almost absent from the recreational market. Instead, the relatively large worldwide diffusion of the most potent 3-MeO-PCP represents the cause of many non-fatal and fatal intoxication cases (Stevenson and Tuddenham, 2014; Bäckberg et al., 2015a; Bakota et al., 2016; Bertol et al., 2017; Johansson et al., 2017; Mitchell-Mata et al., 2017; Zidkova et al., 2017; Ameline et al., 2019b; Ameline et al., 2019c; Berar et al., 2019; de Jong et al., 2019; Grossenbacher et al., 2019; Kintz et al., 2019).

Widespread approaches to identification of NPS rely on gas chromatography (GC) or liquid chromatography (LC) separation coupled to mass spectrometry (MS) detection. A major challenge is thus posed by discrimination between regioisomers, in cases where the compounds show identical retention behavior, as well as fragmentation patterns. The combined use of different techniques may be required to gather complementary data supporting the submitted case. In a very recent report on a fatal intoxication case, the simultaneous combination of nuclear magnetic resonance (^1^H NMR), ultra-high-performance liquid chromatography-tandem mass spectrometry (UHPLC-MS/MS), and GC-MS was deemed as necessary to discriminate the structural isomers of MeO-PCP in the biological specimens of the victim (Berar et al., 2019).

Likewise, Fourier Transform Infrared Spectroscopy (FTIR) can be very specific to the determination of functional groups within unknown samples by measuring small energy differences based on rotational and vibrational amplitudes between individual molecular bonds. In this regard, GC-FTIR data may well complement the information afforded by GC-MS and LC-MS in discriminating between regioisomeric compounds (Lee et al., 2019). Especially on-line techniques relying on the use of direct deposition interfaces enable sensitivity of at least two orders of magnitude more (at the ng scale) than the gas phase devices. Moreover, the sharper IR absorption bands arising from analytes in the solid state result in a significant gain in resolution with respect to spectra acquired from gas molecules (down to 4 cm^−1^) affected by centrifugal distortion (Schneider and Demirgian, 1986). The usefulness of solid deposition GC-FTIR (sd-GC-FTIR) in forensic laboratories has been demonstrated already for a number of different drug categories contained in seized drugs (Lanzarotta et al., 2017; Cheng and Wong, 2019; Salerno et al., 2020); however the characterization of unknown molecules in biological specimens has never been attempted.

In this paper, we describe the analytical characterization, following two non-fatal intoxication cases, of 3-MMC and 3-MeO-PCP in seized products, and 3-MeO-PCP and its metabolites in biosamples. Different analytical techniques were employed, i.e., GC-MS with electron impact ionization (GC-EI MS), liquid chromatography-high-resolution accurate-mass Orbitrap mass spectrometry (LC-HRAM-Orbitrap-MS), and sd-GC-FTIR. The role of the two latter techniques in attaining full structural characterization of the psychoactive drugs and related metabolites, in both non-biological and biological samples, is highlighted.

## Materials and Methods

### Chemicals

Water, acetonitrile, formic acid, ethyl acetate, methanol, and 3,4-Methylenedioxy-N-propylamphetamine (MDPA, internal standard, IS) were purchased from Sigma-Aldrich (Milan, Italy); ammonium formate, potassium dihydrogen phosphate, sodium hydroxide, and Bond Elute Certify 130 mg Solid Phase Extraction (SPE) columns were obtained from Agilent Technologies (Santa Clara, CA, United States).

### Sample Preparation

#### Seized Powders

Two white powders, contained in small bags, were seized by the police from two subjects later admitted to two nearby emergency departments because of neurological impairment. Bag labels indicated 3-MMC and 3-MeO-PCP as active ingredients. Police investigations found that both powders were purchased online. For analytical determination, 1 mg of each seized products were dissolved in 1 mL of methanol. About 1 μL of a one-tenth-fold diluted methanol solution was injected into the GC–MS and sd-GC-FTIR systems, whereas 10 μL of a 100-fold diluted methanol:LC mobile phase A (water with 0.05% formic acid and 10 mM ammonium formate) (10:90, v/v) solution were injected in the LC-HRAM-Orbitrap-MS system.

#### Biological Fluids

An immunoassay toxicological screening for common drugs of abuse and an enzymatic ethanol test were performed in urine and serum samples of both subjects upon hospital admission. For further toxicological tests, whole blood and urine samples were collected from subject A on admission, while plasma and urine samples were collected from subject B when admitted, and whole blood and urine the day after. For LC-HRAM-Orbitrap-MS analysis, 50 µL aliquots of a 2 μg/mL MDPA methanolic solution were poured in conical bottom tubes and evaporated to dryness by gently blowing nitrogen at an ambient temperature. Whole blood, plasma, and urine samples (250 µL each) were subjected to protein precipitation by adding 750 μL of acetonitrile/methanol 2:1 (v/v), previously stored at 4°C, drop by drop while vortexing for 20 s. Precipitated samples were centrifuged at 2,800 rpm for 5 min and the supernatants taken to dryness. The residues were reconstituted with 50 μL aliquots of methanol:LC mobile phase A (10:90, v/v) and transferred to glass inserts contained in 2 mL autosampler vials. Ten μL aliquots of the obtained solutions were injected onto the LC-HRAM-Orbitrap-MS system. For sd-GC-FTIR analysis, urine samples (0.5 mL each) were diluted with 6 mL of 0.1 M phosphate buffer and extracted on Bond Elute Certify 130 mg SPE columns. The obtained eluates were taken to dryness by gently blowing nitrogen at ambient temperature. The residues were reconstituted with 15 μL of methanol and transferred to glass inserts contained in 2 mL autosampler vials. One μL aliquots of the methanolic solutions were injected into the sd-GC-FTIR system.

### GC-EI MS

GC-EI MS analyses of the seized powders were carried out as previously published (Frison et al., 2016b). Briefly, an Agilent 7890 series II/5975 GC/MS quadrupole mass spectrometer operating with electron ionization (EI, 70 eV) in full scan (*m*/*z* 40–600) acquisition mode (Agilent Technologies, Cernusco sul Naviglio, Italy) was used. The capillary column was an Agilent HP-5MS UI (ultra inert, 30 m × 0.25 mm, 0.25 μm film thickness), and the oven temperature was programmed from 50 to 300°C. Compounds were identified by comparison within the SWGDRUG MS Library version 3.6 (available at http://www.swgdrug.org/ms.htm), containing over 3,000 EI mass spectra of drugs and drug-related compounds.

### LC-HRAM-Orbitrap-MS

LC-HRAM-Orbitrap-MS analyses of both non-biological and biological specimens were carried out employing the analytical conditions previously published for the characterization of several NPS (Frison et al., 2015; Frison et al., 2016a; Frison et al., 2016b; Papa et al., 2019). Briefly, powder solutions and biosample extracts were analyzed using a Thermo Scientific Accela 1250 UHPLC system equipped with a Hypersil Gold PFP analytical column (2.1 mm × 100 mm, 1.9 µm particle size), coupled to a Thermo Scientific single-stage Exactive HCD MS system, interfaced with an HESI-II source. Mobile phase A was water with 0.05% formic acid and 10 mM ammonium formate, and mobile phase B was acetonitrile with 0.05% formic acid. The flow rate was set to 400 μL/min. The mobile phase gradient was as follows: 99% A for 1 min, linear gradient to 70% B in 6.5 min, linear gradient to 100% B in 1 min, held for 5.0 min, and column re-equilibration was performed with linear gradient to 99% A in 0.5 min, held for 3.0 min. MS was performed in positive-ion mode with a scan range from *m*/*z* 50 to 800, alternating full scan (HCD off, resolution of 100,000 FWHM at *m*/*z* 200) and “all-ion fragmentation” (AIF) (HCD on, collision energies 10, 25, and 50 eV, resolution of 25,000 FWHM at *m*/*z* 200) acquisition. External mass calibration was performed, according to the guidelines provided by the instrument supplier, every 2 days over the mass range *m*/*z* 130–2,000. Data were acquired and processed using Thermo Scientific Excalibur software version 2.1.0.

### sd-GC-FTIR

GC separations of seized powders and biological specimens were performed on a Nexis GC-2030 gas chromatograph equipped with an AOC-20i autosampler (Shimazdu, Kyoto, Japan) coupled to a DiscovIR solid deposition FTIR detector (Spectra-Analysis Instrument Inc., Marlborough, MA, United States). A Supelco SLB-5ms column (30 m × 0.25 mm, 0.25 μm film thickness) was employed for the separation (Merck KGaA, Darmstadt, Germany), using helium (purity 99.99%) as a carrier gas, at a constant linear velocity of 30 cm/s. The 1 μL samples were injected in splitless mode (1.50 min sampling time) at an injector temperature of 280°C. The oven temperature program was set to 100°C for 2 min before being ramped to 350°C at 15°C/min. The final temperature was held for 5.0 min, resulting in total analysis times of 24.0 min.

The end of the column was connected to a deactivated fused silica capillary through a micro Siltite *µ*-union (Trajan Scientific and Medical, Milton Keynes, United Kingdom) and inserted into a heated transfer line connected to the IR interface. The transfer line and restrictor temperatures were set at 280°C. Blanks were run in between samples to assure that the liner and column were free of contamination. The restrictor was positioned directly above a ZnSe disk, which was chilled down to −50°C by means of liquid nitrogen, and cleaned daily with acetone. The DiscovIR FTIR instrument was equipped with a Mercury-Cadmium-Telluride (MCT) cryogenically cooled detector. Solid-phase IR spectra of the GC eluted compounds were recorded in real time from 100 μm × 100 μm spots in the 650–4,000 cm^−1^ range, with a resolution of 4 cm^−1^ and a scan rate of 2 Hz, at a disc rotation speed of 3 mm/min. GC-FTIR data acquisition and processing were performed using the Thermo Galactic GRAMS/AI (version 9.3) spectroscopy and chromatography software. Compounds were identified through the library search program (Spectral ID), using a first derivative correlation algorithm, by comparison within a homemade solid deposition IR spectral library containing IR spectral data of around 600 NPS (namely, Controlled and Prohibited Substances version 1.0).

## Results and Discussion

Identification of synthetic cathinone and PCP analogs, and in particular identification of positional isomers of the MMC and MeO-PCP families, may be particularly challenging for clinical and forensic laboratories. MMC and MeO-PCP analogs may show quite similar chromatographic and, especially, mass spectrometric behaviors, which may hinder their full characterization in the case of unavailability of reference standards for all isomers, and/or their metabolites in case of biosample analysis. These analytical challenges have been addressed by several authors by means of different analytical approaches, for the discrimination of 2-MMC, 3-MMC, and 4-MMC (Power et al., 2011; Power et al., 2012; Jamey et al., 2016; Maas et al., 2017; Zuba and Adamowicz, 2017; Nowak et al., 2018; Kranenburg et al., 2019; Kranenburg et al., 2020a; Kranenburg et al., 2020b) and 3-MeO-PCP and 4-MeO-PCP (Bäckberg et al., 2015a; Bakota et al., 2016; Bertol et al., 2017; Johansson et al., 2017; Mitchell-Mata et al., 2017; Zidkova et al., 2017; Ameline et al., 2019b; Berar et al., 2019; de Jong et al., 2019; Kintz et al., 2019). However, such discriminative analyses were in most cases carried out with all the necessary reference standards available. We attained the full identification of the two NPS under study, in both non-biological (3-MMC and 3-MeO-PCP) and biological (3-MeO-PCP) specimens, through a combination of GC-EI MS, LC-HRAM-Orbitrap-MS, and sd-GC-FTIR, without resorting to specific reference standards. In particular, reliable results obtained from searching into dedicated spectral libraries allow for confident compound identification to be attained even if the reference material is not at hand.

### Analysis of Seized Powders

**FIGURE 1:**
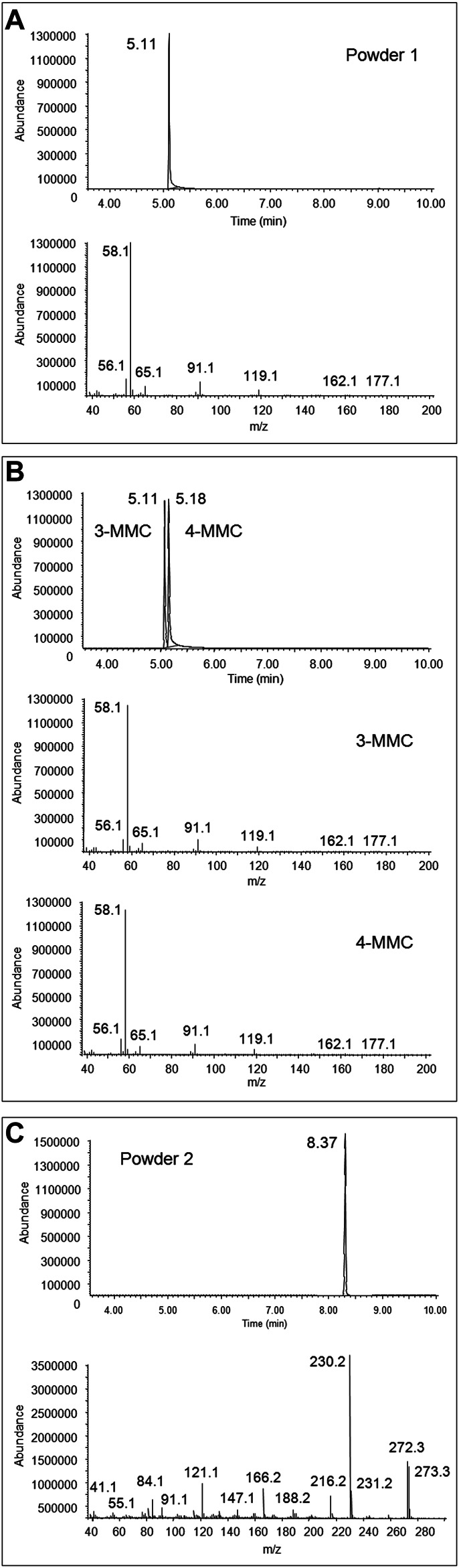
Full scan chromatogram and EI mass spectrum of the peak at 5.11 min obtained from GC-MS analysis of the first seized powder **(A)**, full scan chromatogram and EI mass spectra, obtained from GC-MS analysis of 3-MMC and 4-MMC standards **(B)**, and full scan chromatogram and EI mass spectrum of the peak at 8.37 min, obtained from GC-MS analysis of the second seized powder **(C)**.

3-MMC and 3-MeO-PCP were putatively identified in the two seized powders through comparison of their GC-EI MS spectra with those of the SWGDRUG library and 3-MMC and 4-MMC reference standards, as, at the time of case processing, no 2-MMC and MeO-PCP standards were available (Figure 1). As a matter of fact, the identification of MMC and MeO-PCP positional isomers was not conclusive by GC-MS alone. Actually, isomers of the two chemical families may show similar EI spectra. For instance, 4-MMC, 3-MMC, and 2-MMC EI spectra are hardly distinguishable due to their extreme similarity (Power et al., 2011; Zuba and Adamowicz, 2017); they are in all cases characterized by the barely visible molecular ions at *m*/*z* 177, not abundant fragment ions at *m*/*z* 162, 119, 91, 65, and 56 and the unspecific base peak at *m*/*z* 58, caused by the well-known formation of the immonium ion via the amine-initiated alpha-cleavage of the benzylic bond, characteristic of all N-methyl phenethylamines (Frison et al., 2011). Also, 3-MeO-PCP and 4-MeO-PCP are hardly distinguishable by their EI spectra (Bertol et al., 2017; Mitchell-Mata et al., 2017; Kintz et al., 2019).

**FIGURE 2:**
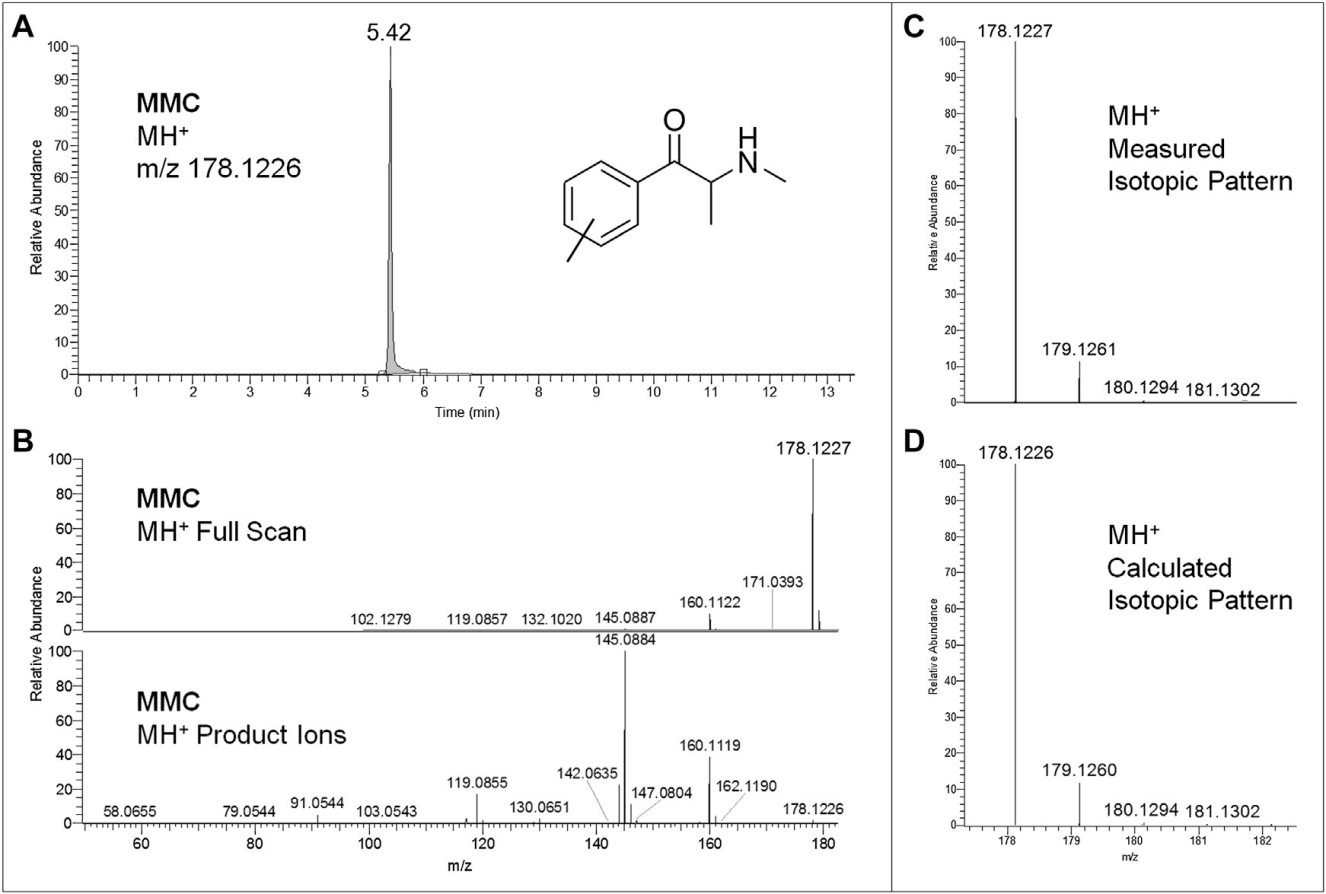
Extracted ion chromatogram at *m*/*z* 178.1226 (MMC MH^+^ ions) of the full scan ion trace without fragmentation **(A)**, corresponding MMC full scan mass spectrum and MH^+^ collision-induced product ion spectrum (collision energy 25 eV) **(B)**, and experimental **(C)** and calculated **(D)** isotopic patterns of MMC MH^+^ ions, all obtained from LC-HRAM-Orbitrap-MS analysis of the first seized powder.

**FIGURE 3:**
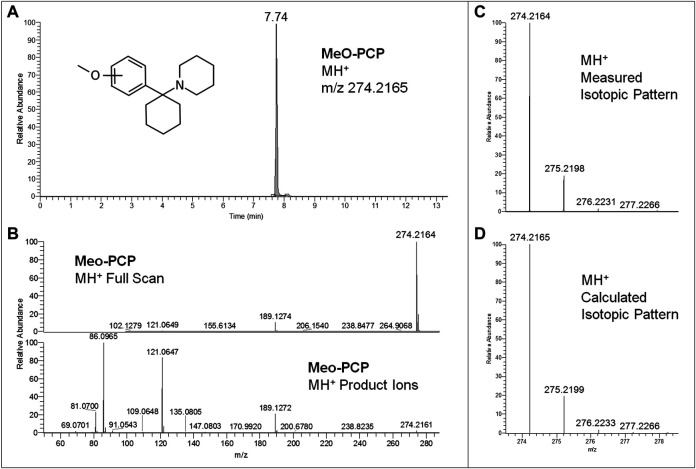
Extracted ion chromatogram at *m*/*z* 274.2165 (MeO-PCP MH^+^ ions) of the full scan ion trace without fragmentation **(A)**, corresponding MeO-PCP full scan mass spectrum and MH^+^ collision-induced product ion spectrum (collision energy 25 eV) **(B)**, and experimental **(C)** and calculated **(D)** isotopic patterns of MeO-PCP MH^+^ ions, all obtained from LC-HRAM-Orbitrap-MS analysis of the second seized powder.

Regarding LC-HRAM-Orbitrap-MS analysis of the two seized powders, Figures 2, 3 show the extracted ion chromatograms at *m*/*z* 178.1226 (MMC MH^+^ ions) and 274.2165 (MeO-PCP MH^+^ ions) of the full scan ion traces without fragmentation, the corresponding MMC and MeO-PCP full scan mass spectra and related MH^+^ collision-induced product ion spectra obtained in AIF conditions (HCD on, 25 eV), as well as the experimental and calculated isotopic patterns of MMC and MeO-PCP MH^+^ ions, obtained from the LC-HRAM-Orbitrap-MS analysis of the first and the second seized powder, respectively. Accurate mass measurements of the two MH^+^ ions had a mass accuracy [Δm = (accurate mass − exact mass)/exact mass × 10^6^] of +0.56 and −0.36 ppm for MMC and MeO-PCP, respectively. Fully superimposable experimental and calculated MH^+^ isotopic patterns were obtained for the two substances, with relative isotopic abundance (RIA, M+1/M+0 ion abundances) error values (expressed as experimental RIA–calculated RIA)/calculated RIA×100) of −4.11 and −2.18% for MMC and MeO-PCP, respectively. Hence, LC-HRAM-Orbitrap-MS measurements allowed us to assign the elemental formulae C_11_H_15_NO (MMC) and C_18_H_27_NO (MeO-PCP) for the two MH^+^ ions. However, MH^+^ collision-induced product ions spectra, although in agreement with those already described (Bakota et al., 2016; Frison et al., 2016a; Michely et al., 2017; Zidkova et al., 2017), were once again not conclusive in discriminating between the positional isomers [(3-MMC vs. 4-MMC and 2-MMC) and (3-MeO-PCP vs. 4-MeO-PCP)] without using reference standards, as even in these analytical conditions fragment ions sporting the same mass and similar abundances may be obtained from MH^+^ ions of individual MMC (Maas et al., 2017; Zuba and Adamowicz, 2017) and MeO-PCP (Bakota et al., 2016; Bertol et al., 2017; Zidkova et al., 2017) analogs.

**FIGURE 4:**
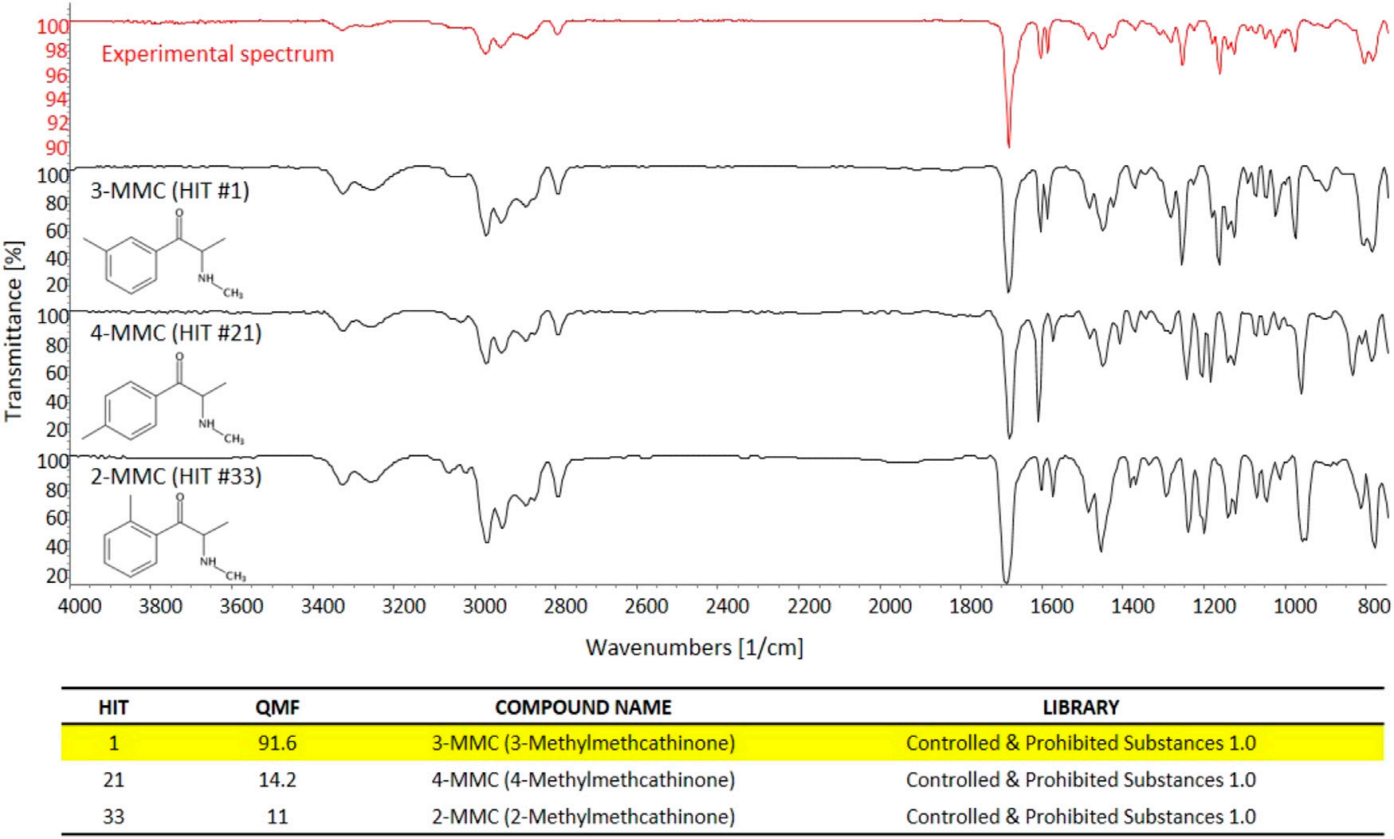
The experimental GC-FTIR spectrum of 3-MMC, compared to the library GC-FTIR spectra of 3-MMC, 2-MMC, and 4-MMC isomers. Based on the library search results for 3-MMC FTIR spectral data, the following QMF values were obtained against correct and incorrect matches: Hit #1, 91.6%; Hit #21, 14.2%; Hit #33, 11.0%.

Conversely, sd-GC-FTIR allowed us to identify MMC and MeO-PCP positional isomers unambiguously, confirming the presence of 3-MMC in the first seized powder and 3-MeO-PCP in the second seized powder. Confident discrimination of the positional isomers was possible based on the high quality match factor (QMF) of the experimental FTIR spectra against the target FTIR spectra upon searching in a dedicated solid-phase GC-FTIR library. The sharp absorption bands in FTIR spectra obtained from solid deposited analytes allow us to easily differentiate between very similar compounds, as shown in Figure 4 for 3-MMC, 4-MMC, and 2-MMC. For identification purposes, the experimental GC-FTIR spectrum obtained for the cathinone compound contained in the first seized powder was searched into a solid-phase GC-FTIR library containing IR spectral data of NPS (supplied by the vendor). A QMF criterion was adopted, based on quantitative evaluation of IR spectral data, as an unbiased criterion to achieve unambiguous compound identification. Thus, 3-MMC could be identified in the sample, ranking as Hit #1 in the library search result, with a QMF of 91.6%. Noticeably, much lower QMF values were obtained for the two incorrect matches, *viz*. 14.2% for the 4-substituted cathinone (4-MMC) and 11.0% for the 2-substituted isomer (2-MMC). It is also worth underlining that 4-MMC did not rank in the second hit position, as one could expect, but showed up as Hit #21, after a long list of candidates, and isomer 2-MMC was Hit #33.

**FIGURE 5:**
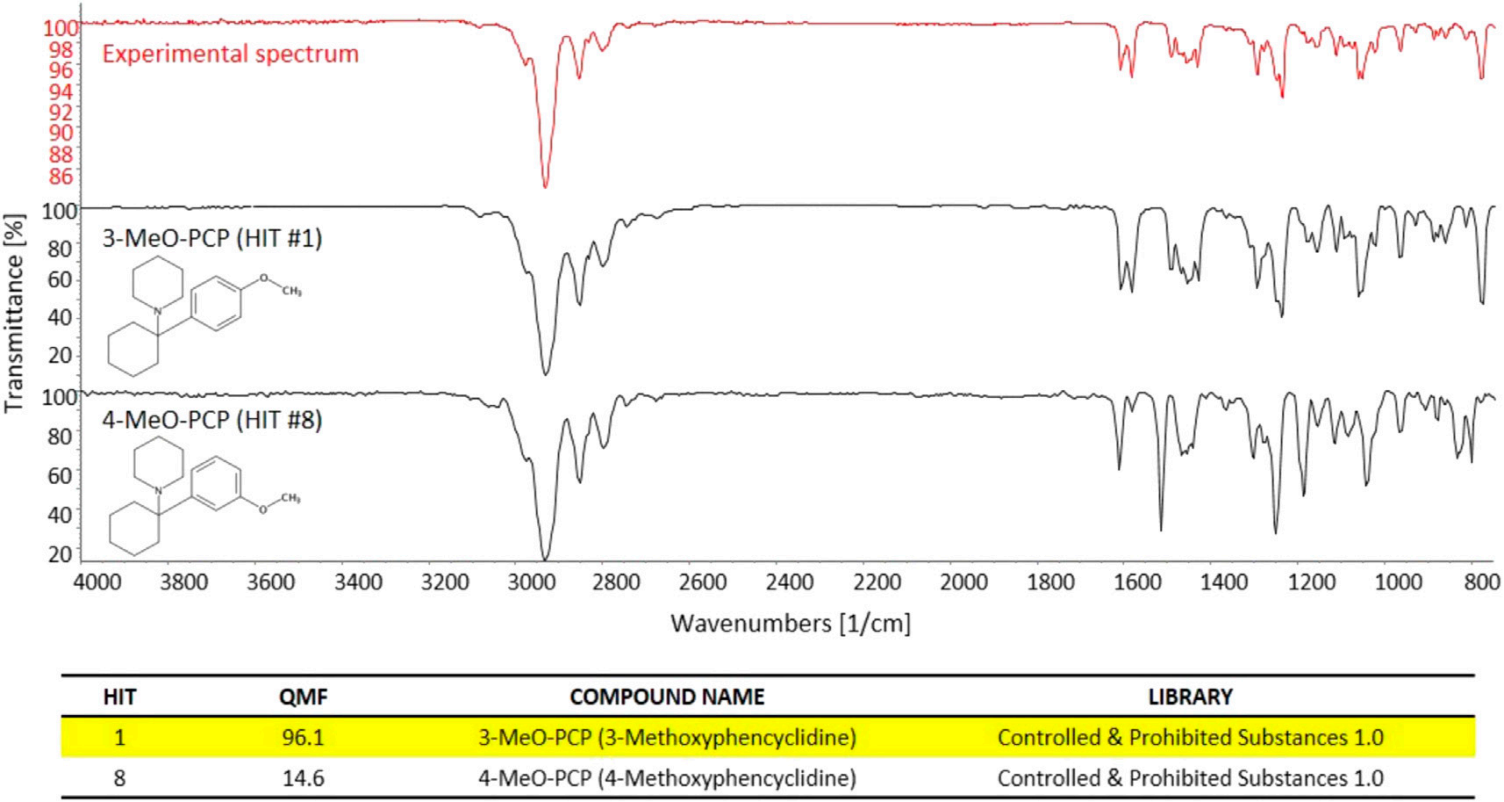
The experimental GC-FTIR spectrum of 3-MeO-PCP, compared to the library GC-FTIR spectrum of 3-MeO-PCP and 4-MeO-PCP isomers. Based on the library search results for 3-MMC FTIR spectral data, the following QMF values were obtained against correct and incorrect match: Hit #1, 96.1%; Hit #8, 14.6%.

In a similar way, the great specificity of molecule/bond-related information contained in the GC-FTIR spectrum allowed for a definite identification of the illicit drug constituent of the second seized powder, i.e., 3-MeO-PCP. Again, a high QMF differential was obtained between the correct and the (closest) incorrect match, i.e., the 3-methoxy-substituted phencyclidine and the 4-substituted isomer, thanks to distinctive spectral features especially in the fingerprint region to the right hand side of Figure 5 (below 1,500 cm^−1^). In detail, 3-MeO-PCP was found as Hit #1, with a QMF of 96.1%, while 4-MeO-PCP showed up as Hit #8, and with only 14.6% of a QMF. The significant gap in library search results would in turn reduce the likelihood for false positives and increase confidence in the identification. In more detail, the compounds that match in the library search between the 1st and 21st hit for MMC were as follows: 3-Methylmethcathinone (Hit 1), 3-Methylethcathinone (Hit 2), 3-Ethylmethcathinone (Hit 3), Pentedrone (Hit 4), N-Methylbenzedrone (Hit 5), 4-Chloropentedrone (Hit 6), Ethcathinone (Hit 7), N-Ethylbuphedrone (Hit 8), 4-Methylcathinone (Hit 9), Cathinone (Hit 10), 3′,4′-Tetramethylene-alpha-pyrrolidinovalerophenone (Hit 11), 4-Bromomethcathinone (Hit 12), α-Isopropylamino-valerophenone (Hit 13), 4-Ethylmethcathinone (Hit 14), 3-Fluoromethcathinone (Hit 15), N-Ethylhexedrone (Hit 16), 4-Methyldiethcathinone (Hit 17), 3′,4′-Tetramethylene-α-pyrrolidinobutiophenone (Hit 18), 4-Fluorobuphedrone (Hit 19), 4- Chloroethcathinone (Hit 20), and 4-Methylmethcathinone (Hit 21). The compounds that match in the library search between the first and eighth hit for MeO-PCP were as follows: 3-Methoxyphencyclidine (Hit 1), 3-Methoxyeticyclidine (Hit 2), Cannabicyclohexanol (Hit 3), Diphenidine (Hit 4), 4-[1-(3-Methoxyphenyl)cyclohexyl]morpholine (Hit 5), 5b-Androstane-3a,17b-diol (Hit 6), 4′-Methyl-2-piperidinopropiophenone (Hit 7), and 4-Methoxyphencyclidine (Hit 8).

### Analysis of Biological Fluids

All immunoassay toxicological screening for common drugs of abuse (not including PCP) performed in urine and serum samples were negative, which is the same as what occurred in other cases (Berar et al., 2019). If the immunoassay screening had included the PCP test (not a routine test in most European toxicology laboratories), a positive result would probably have been obtained considering the cross-reactivity of MeO-PCP analogs in several PCP immunoassay systems (Bäckberg et al., 2015a; Bakota et al., 2016; Chang and Smith, 2017; Mitchell-Mata et al., 2017; Gomila et al., 2019; Larabi et al., 2019b). Enzymatic ethanol tests were also negative in both cases.

Whole blood and urine samples collected from subject A, as well as whole blood, plasma, and urine samples collected from subject B on two consecutive days, were subjected to LC-HRAM-Orbitrap-MS analysis following a simple and rapid deproteinization step. 3-MeO-PCP was detected in all biosamples of both subjects, while no 3-MMC nor its metabolites dihydro-3-MMC (3-methylephedrine), nor-dihydro-3-MMC (3-methylnorephedrine), nor-3-MMC, hydroxytolyl-3-MMC, nor-hydroxytolyl-3-MMC, carboxy-3-MMC, carboxy-dihydro-3-MMC (Pedersen et al., 2013; Frison et al., 2016a) were detected in the samples from both subjects. This suggests that, of the two powders seized by police, only the one containing 3-MeO-PCP had been taken. No other NPS, traditional illicit drugs, or medications (other than those administered at the hospitals) were detected in both cases.

In addition, the comprehensive, untargeted, and sensitive analytical approach, consisting of generic sample preparation (a simple deproteinization step) and full scan MS detection in the high-resolution mode, allowed us to detect more than 20 phase I and 10 phase II 3-MeO-PCP metabolites in all biological specimens. Identification of metabolites was based on the following: 1) evaluation of their chromatographic behavior compared to the parent compound; 2) accurate mass measurements of their MH^+^ ions in full scan conditions; 3) evaluation of their MH^+^ isotopic patterns, including mass accuracy of MH^+^ (M+0, M+1, M+2, M+3) isotopic peaks; 4) accurate mass measurements of MH^+^ collision-induced productions (at three collision energies, 10, 25, 50 eV); and, above all, 5) comparison with analytical results obtained with similar LC-HRAM-Orbitrap-MS instruments, reported in recently published papers dealing with 3-MeO-PCP metabolic studies on rat urine and human liver preparations (Michely et al., 2017), and a molecular networking approach application in a case of 3-MeO-PCP intoxication (Allard et al., 2019). In these studies, numerous phase I metabolites (related to 3-MeO-PCP multiple aliphatic hydroxylations at the cyclohexyl ring and the heterocyclic ring, single aromatic hydroxylation, carboxylation after ring opening, O-demethylation) and corresponding phase II glucuronide metabolites have been identified.

**FIGURE 6:**
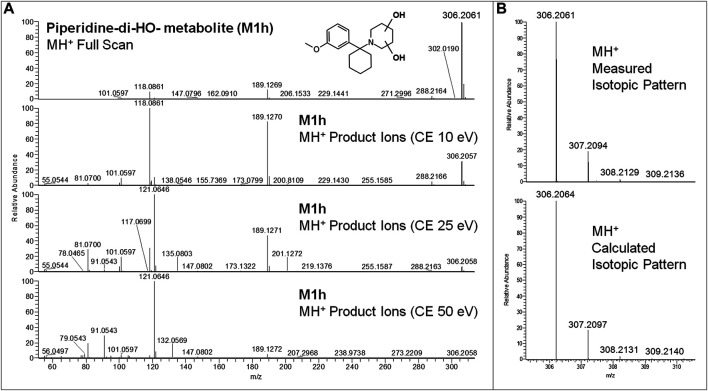
3-MeO-PCP piperidine-di-OH-metabolite (M1h) LC-HRAM-Orbitrap-MS full scan mass spectrum and MH^+^ collision-induced product ion spectra (collision energies 10, 25, and 50 eV) **(A)**, experimental and calculated isotopic patterns of M1h MH^+^ ions **(B)**, all obtained from LC-HRAM-Orbitrap-MS analysis of urine samples.

**FIGURE 7:**
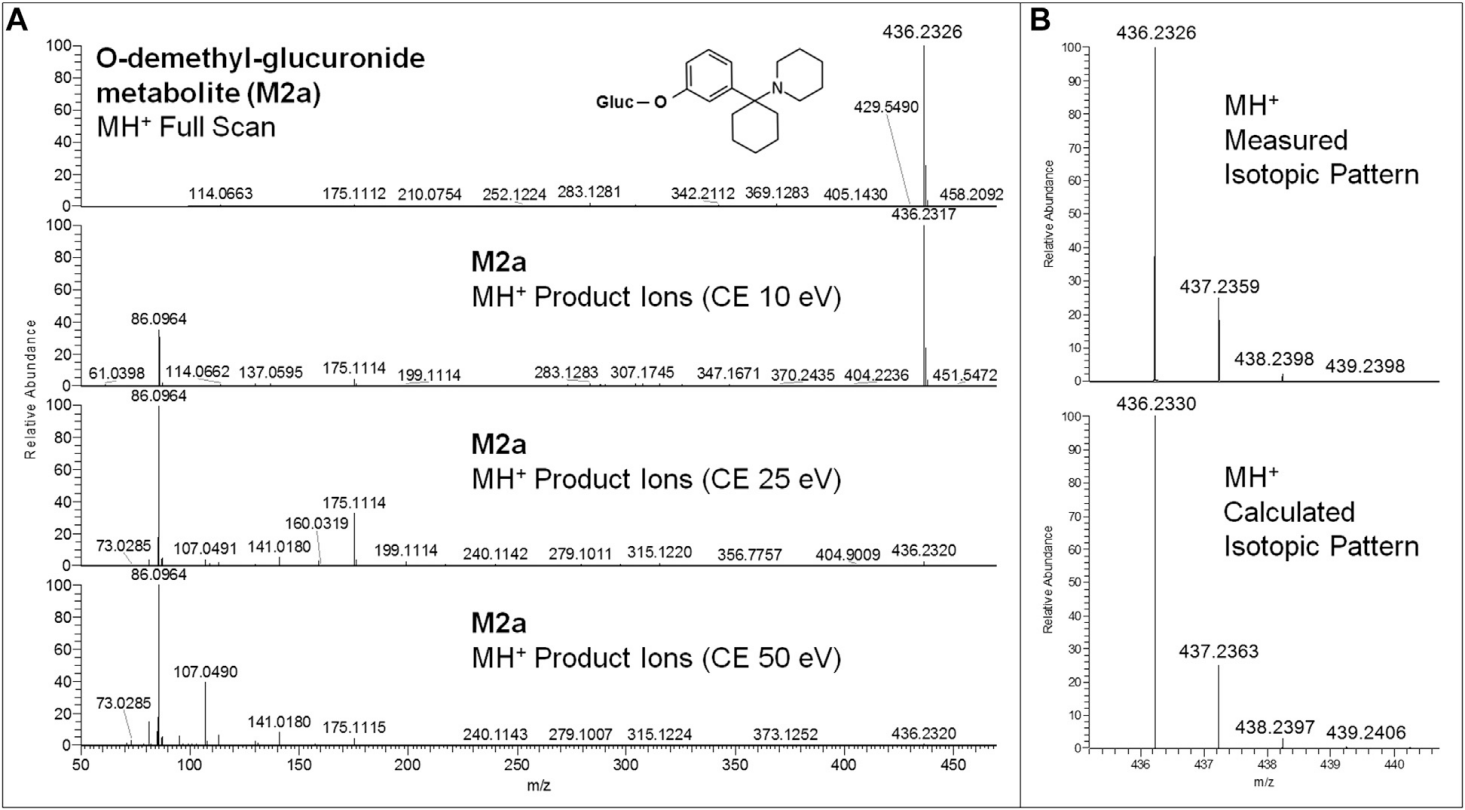
3-MeO-PCP O-demethyl-glucuronide-metabolite (M2a) LC-HRAM-Orbitrap-MS full scan mass spectrum and MH^+^ collision-induced product ion spectra (collision energies 10, 25, 50 eV) **(A)**, and experimental and calculated isotopic patterns of M2a MH^+^ ions **(B)**, all obtained from LC-HRAM-Orbitrap-MS analysis of urine samples.

All equivalent analytical data from the present study are listed in detail in Table 1, as they may become supposedly helpful to other toxicologists in case of further 3-MeO-PCP intoxication cases. All phase I and phase II metabolites, being more hydrophilic and polar, elute at shorter retention times than the parent compound under the reported LC conditions, as previously described (Michely et al., 2017; Zidkova et al., 2017; Allard et al., 2019; Ameline et al., 2019c). Experimental isotopic patterns of each metabolite were in total agreement with the theoretical ones [MH^+^ (M+0) Δm values range from 0 to 1.37 ppm; (M+1) values from 0 to 1.19 ppm; (M+2) values from 0 to 2.76 ppm; (M+3) values from −0.82 to 4.20 ppm], and MH^+^ fragment ions, identified in variable numbers for each metabolite, mirrored those described in the above papers. Some values, mainly related to fragment ions, are missing due to the very low abundance of the specific metabolites. Typical full scan mass spectra, MH^+^ collision-induced product ion spectra obtained applying the three different (10, 25, 50 eV) collision energies, as well as experimental and calculated isotopic patterns, related to one phase I [piperidine-di-OH- (M1h)] and one phase II [O-demethyl-glucuronide- (M2a)] urinary metabolites, are shown in Figures 6 and 7. Similar results are shown in the Supplementary Figures S1 and S2 for the O-demethyl- (M1a), O-demethyl-piperidine-OH- (M1b), and O-demethyl-piperidine-di-OH- (M1g) phase I metabolites as well as the O-demethyl-piperidine-OH-glucuronide- (M2c) phase II metabolite.

**TABLE 1:** Chromatographic and mass spectrometric data of 3-MeO-PCP and its phase I and phase II metabolites identified in the biological specimens under study.

3-MeO-PCP Metabolite(s)	Acronym	MH^+^ elemental composition	*t*_*R*_ (min)	Exact mass, accurate mass, mass accuracy				MH^+^ product ions						
				M+0 (MH^+^)	M+1	M+2	M+3	n. 1	n. 2	n. 3	n. 4	n. 5	n. 6	n. 7
3-MeO-PCP	3-MeO-PCP	C_18_H_28_NO	7.74	274.2165 274.2164 −0.36 ppm	275.2199 275.2198 −0.36 ppm	276.2233 276.2231 −0.72 ppm	277.2266 277.2266 0 ppm	189.1272	121.0647	95.0492	91.0543	86.0965	81.0700	-
O-demethyl-	M1a	C_17_H_26_NO	6.42	260.2009 260.2008 −0.38 ppm	261.2042 261.2041 −0.38 ppm	262.2076 262.2074 −0.76 ppm	263.2110 263.2109 −0.38 ppm	175.1115	107.0491	95.0492	91.0542	86.0965	81.0700	79.0544
O-demethyl-piperidine-OH-	M1b	C_17_H_26_NO_2_	5.49	276.1958 276.1956 −0.72 ppm	277.1992 277.1990 −0.72 ppm	278.2025 278.2023 −0.72 ppm	279.2059 279.2061 0.72 ppm	175.1113	107.0490	102.0912	95.0491	84.0808	81.0699	77.0388
O-demethyl-cyclohexyl-HO-	M1c	C_17_H_26_NO_2_	4.55 5.01* 5.09	276.1958 276.1958 0 ppm	277.1992 277.1992 0 ppm	278.2025 278.2025 0 ppm	279.2059 279.2057 −0.72 ppm	173.0959	107.0491	86.0966	79.0544	77.0387	-	-
O-demethyl-cyclohexyl-HO-dehydro-oxo-piperidine-	M1d	C_17_H_22_NO_3_	5.30	288.1594 288.1592 −0.69 ppm	289.1628 289.1625 −1.04 ppm	290.1661 290.1653 −2.76 ppm	-	98.0601	-	-	-	-	-	-
Piperidine-HO-	M1e	C_18_H_28_NO_2_	6.68	290.2115 290.2112 −1.03 ppm	291.2148 291.2146 −0.69 ppm	292.2182 292.2180 −0.68 ppm	293.2215 293.2211 −1.36 ppm	189.1274	121.0648	102.0914	91.0543	84.0809	81.0701	-
Cyclohexyl-HO- isomers	M1f	C_18_H_28_NO_2_	5.62* 6.18 6.34	290.2115 290.2112 −1.03 ppm	291.2148 291.2146 −0.69 ppm	292.2182 292.2179 −1.03 ppm	293.2215 293.2211 −1.36 ppm	205.1221	187.1116	121.0647	91.0543	86.0965	79.0544	77.0387
O-demethyl-piperidine-di-HO-	M1g	C_17_H_26_NO_3_	5.88	292.1907 292.1903 −1.37 ppm	293.1941 293.1938 −1.02 ppm	294.1974 294.1971 −1.02 ppm	295.1983 295.1977 −2.03 ppm	175.1114	118.0860	107.0490	101.0597	81.0699	79.0544	77.0387
Piperidine-di-HO-	M1h	C_18_H_28_NO_3_	6.95	306.2064 306.2061 −0.98 ppm	307.2097 307.2094 −0.98 ppm	308.2131 308.2129 −0.65 ppm	309.2140 309.2136 −1.29 ppm	189.1270	121.0646	118.0861	101.0597	100.0757	91.0543	81.0700
Cyclohexyl-HO-piperidine-HO- isomers	M1i	C_18_H_28_NO_3_	4.45* 5.15 5.33	306.2064 306.2063 −0.33 ppm	307.2097 307.2096 −0.33 ppm	308.2131 308.2131 0 ppm	309.2140 309.2127 −4.20 ppm	187.1114	121.0647	102.0914	84.0808	-	-	-
O-demethyl-cyclohexyl-HO-piperidine-di-HO-	M1k	C_17_H_26_NO_4_	4.72	308.1856 308.1856 0 ppm	309.1890 309.1889 −0.32 ppm	310.1923 310.1921 −0.64 ppm	-	191.1064	173.0958	118.0861	101.0597	-	-	-
Carboxy- methyl artifact	M1m	C_19_H_30_NO_3_	7.86	320.2220 320.2217 −0.94 ppm	321.2254 321.2251 −0.93 ppm	322.2287 322.2285 −0.62 ppm	323.2296 323.2297 0.31 ppm	189.1271	132.1017	115.0753	-	-	-	-
O-(demethyl-carboxy-cyclohexyl-HO-) methyl artifact	M1n	C_18_H_28_NO_4_	5.73	322.2013 322.2010 −0.93 ppm	323.2046 323.2044 −0.62 ppm	324.2080 324.2075 −1.54 ppm	-	-	-	-	-	-	-	-
Cyclohexyl-OH-piperidine-di-OH-	M1o	C_18_H_28_NO_4_	5.04	322.2013 322.2012 −0.31 ppm	323.2046 323.2045 −0.31 ppm	324.2080 324.2081 0.31 ppm	325.2089 325.2079 −3.07 ppm	187.1115	118.0860	100.0756	-	-	-	-
Carboxy-cyclohexyl-HO- methyl artifact isomers or Carboxy-alkyl-OH-	M1p	C_19_H_30_NO_4_	7.52 7.69* 8.23	336.2169 336.2166 −0.89 ppm	337.2203 337.2199 −1.19 ppm	338.2236 338.2230 −1.77 ppm	-	-	-	-	-	-	-	-
O-demethyl- glucuronide	M2a	C_23_H_34_NO_7_	4.47	436.2330 436.2326 −0.92 ppm	437.2363 437.2359 −0.91 ppm	438.2397 438.2398 0.23 ppm	439.2406 439.2398 −1.82 ppm	175.1114	107.0490	95.0128	86.0964	81.0700	79.0544	-
O-demethyl-aryl-OH glucuronide	M2b	C_23_H_34_NO_8_	3.41	452.2279 452.2274 −1.11 ppm	453.2312 453.2310 −0.44 ppm	454.2346 454.2346 0 ppm	455.2355 455.2344 −2.42 ppm	191.1063	173.0958	107.0490	86.0964	79.0543	-	-
O-demethyl-piperidine-OH- glucuronide	M2c	C_23_H_34_NO_8_	4.07	452.2279 452.2275 −0.88 ppm	453.2312 453.2310 −0.44 ppm	454.2346 454.2346 0 ppm	455.2355 455.2346 −1.98 ppm	175.1114	107.0490	102.0913	84.0808	81.0699	-	-
O-demethyl-aryl-OH-dihydropyridine-OH- glucuronide	M2d	C_23_H_30_NO_9_	4.20	464.1915 464.1914 −0.22 ppm	465.1949 465.1947 −0.43 ppm	466.1982 466.1982 0 ppm	467.1991 467.1987 −0.86 ppm	173.0958	98.0601	79.0545	-	-	-	-
Piperidine-OH glucuronide	M2e	C_24_H_36_NO_8_	5.54	466.2435 466.2429 −1.29 ppm	467.2469 467.2465 −0.86 ppm	468.2503 468.2501 −0.43 ppm	469.2511 469.2501 −2.13 ppm	278.1227	189.1270	121.0646	102.0913	85.0284	84.0808	81.0700
O-demethyl-cyclohexyl-HO-piperidine-HO- glucuronide	M2f	C_23_H_34_NO_9_	3.77	468.2228 468.2226 −0.43 ppm	469.2262 469.2258 −0.85 ppm	470.2295 470.2294 −0.21 ppm	471.2304 471.2298 −1.27 ppm	-	-	-	-	-	-	-
O-demethyl-piperidine-di-OH- glucuronide	M2g	C_23_H_34_NO_9_	4.44	468.2228 468.2225 −0.64 ppm	469.2262 469.2259 −0.64 ppm	470.2295 470.2293 −0.43 ppm	471.2304 471.2295 −1.91 ppm	145.0657	107.0490	84.0808	79.0543	-	-	-
Cyclohexyl-HO-piperidine-HO- glucuronide	M2h	C_24_H_36_NO_9_	4.53	482.2385 482.2382 −0.62 ppm	483.2418 483.2416 −0.41 ppm	484.2452 484.2449 −0.62 ppm	485.2461 485.2465 0.82 ppm	187.1115	159.0802	121.0647	102.0914	84.0809	-	-
Piperidine-di-OH- glucuronide	M2i	C_24_H_36_NO_9_	5.93	482.2385 482.2379 −1.24 ppm	483.2418 483.2414 −0.83 ppm	484.2452 484.2449 −0.62 ppm	485.2461 485.2460 −0.21 ppm	121.0646	118.0861	-	-	-	-	-

Mass accuracy (Δm) = (accurate mass − exact mass)/exact mass × 10^6^.

In case of isomers MS data refer to the most abundant one (*).

Missing values (-) in some “isotopic pattern” or “product ion” columns are due to the very low abundance of some metabolites.

**FIGURE 8:**
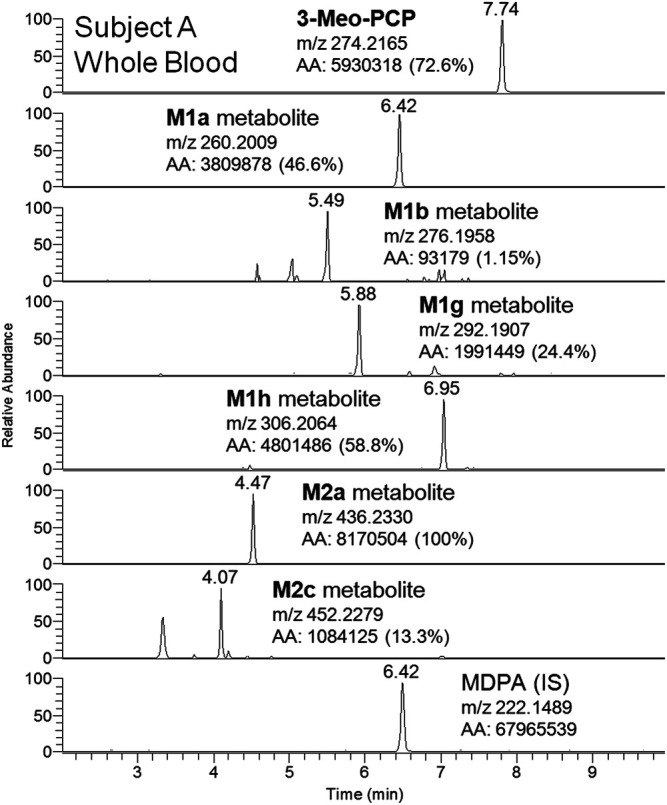
LC-HRAM-Orbitrap-MS ion chromatograms of 3-MeO-PCP, main phase I (M1a, M1b, M1g, and M1h) and phase II (M2a and M2c) metabolites, and the IS MDPA, obtained from whole blood collected at the same time of urine from the same subject.

**FIGURE 9:**
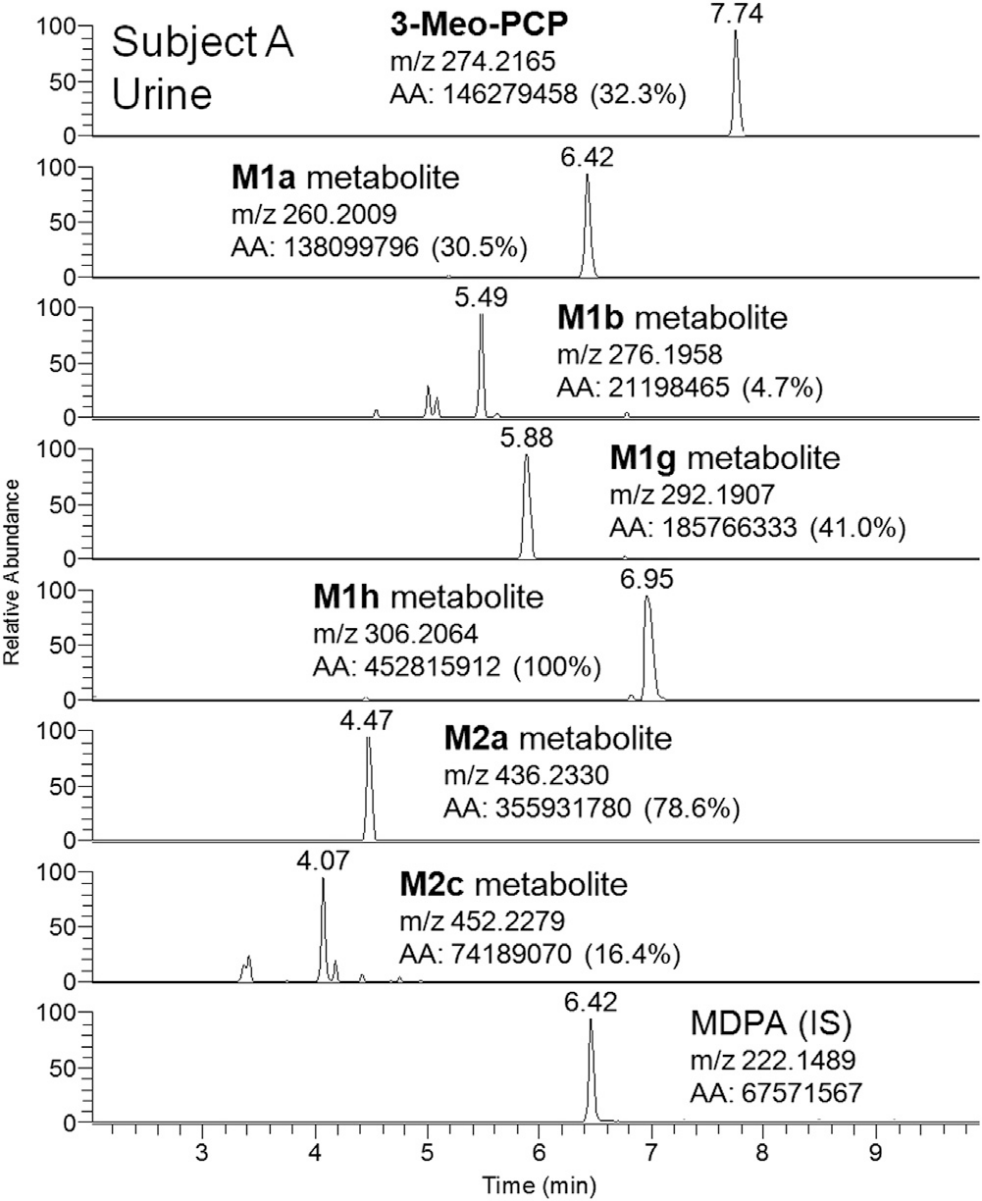
LC-HRAM-Orbitrap-MS ion chromatograms of 3-MeO-PCP, main phase I (M1a, M1b, M1g, and M1h) and phase II (M2a and M2c) metabolites, and the IS MDPA, obtained from urine collected at the same time of whole blood from the same subject.

Although a great number of metabolites have been identified and listed in Table 1, a selection of compounds can be chosen to represent the main targets for an LC-(HR)MS screening of biofluids in case of suspected 3-MeO-PCP intoxication. Therefore, a specific instrumental layout covering the seven most abundant analytes for urine (and whole blood, barring the M1g metabolite) samples was arranged. Urinary metabolites other than the seven chosen all had minor abundances. Figures 8 and 9 show the application of this layout, displaying the corresponding ion chromatograms of 3-MeO-PCP, the chosen phase I (M1a, M1b, M1g, and M1h) and phase II (M2a and M2c) metabolites, and the IS MDPA, obtained from whole blood and urine extracts, respectively, which were collected at the same time from the same subject (subject A in this case). Judging by the absolute and percent area values of compound peaks shown in the Figures, the relative abundances of both whole blood and urine 3-MeO-PCP and metabolite M1h (piperidine-di-OH-) were consistent with corresponding results reported by Allard et al. (2019). Roughly the same, as far as urine is concerned, for the abundance ratio of M1a (O-demethyl-) to M1h (piperidine-di-OH-) metabolites, as well as abundance ratio of M2a (O-demethyl-glucuronide) to M2c (O-demethyl-piperidine-OH-glucuronide) metabolites. Moreover, differently from what is reported by Ameline et al. (2019c), elevated urinary metabolite-to-parent drug ratios were found here for some metabolites (M1g, M1h, and M2a), and this may be considered advantageous to increase the detection windows in intoxication cases. However, this was not the case with subject B, whose day 1 urine showed a higher abundance of parent compound compared to most metabolites. Hence, in these two cases, among other variables, different drug intake times may be hypothesized for the two subjects since, as widely known and already pointed out (Ameline et al., 2019c), metabolite ratios change during the parent drug excretion curve. Also, it should be considered that results from autopsy samples (Ameline et al., 2019c) may not be comparable with results from non-fatal cases, as those described here. As expected, analyte concentrations of biosamples collected from subject B were higher on hospital admission than on day 2 (data not shown). Whole blood 3-MeO-PCP quantitative analysis was not carried out due to 3-MeO-PCP reference standard unavailability at the time of case processing.

**FIGURE 10:**
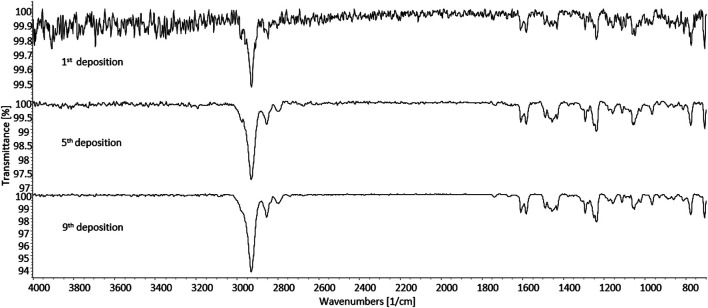
Solid-phase GC-FTIR spectrum of 3-methoxyphencyclidine (3-MeO-PCP) in SPE-extracted human urine sample at 4 cm^−1^ resolution. Top to bottom: increase in the signal-to-noise ratio resulting from multiple deposits of the GC-eluted analyte from consecutive runs overlaid on the disc. Column: Supelco SLB-5ms (30 m × 0.25 mm, 0.25 μm film thickness).

As further identification means, sd-GC-FTIR was applied for the analysis of SPE-extracted urine samples from both subjects. The presence of 3-MeO-PCP was revealed in both cases, thus proving, in conjunction with LC-HRAM-Orbitrap-MS, the intake of such specific methoxy-PCP isomer by both subjects. A first GC-FTIR analysis of urine samples from both subjects did not yield satisfactory results, given the presence of a high number of different metabolites of the parent drug, all showing up as peaks of much higher abundance with respect to the compound under investigation (data shown in Supplementary Figure S3). In accordance with the results from LC-HRAM-Orbitrap-MS, the GC-FTIR peak corresponding to 3-MeO-PCP in a urine sample from patient B revealed much higher intensity than the corresponding one in subject A urine sample, with absorption intensities in the mid-IR region of 0.01 and 0.0032, respectively (data shown in Supplementary Figure S3). In both cases, the amount of analyte being deposited on the IR-transparent disc after GC separation was not sufficient to provide a full informative FTIR spectrum for identification purposes. This was reflected in the low QMF value obtained from searching the experimental spectra in a home-made solid-phase GC-FTIR library, which was equal to 13 for identification of 3-MeO-PCP in a urine sample from subject A. In order to enhance analyte detectability and further obtain confident identification of the target compound, concentrated deposits were obtained through multiple sample deposition. As expected, multiple deposits of the GC-eluted analyte from consecutive runs overlaid on the disc, afforded an increase in the signal-to-noise ratio, as shown in Figure 10. The similarity score, in terms of QMF, has increased to 80 after five consecutive depositions of the analyte, and progressively reached a value >90 after the ninth deposition was accomplished (data shown in Supplementary Figure S4). This is related to a key validation parameter in spectroscopic measurements, defined as the limit of identification (LOI), and representing the lowest analyte concentration that yields a library searchable IR spectrum. Beyond the more common concept of Limit of Detection (LOD), LOI ultimately defines quantitatively the possibility for reliable identification of an unknown compound, contained at a certain amount in a given sample, and often in the presence of a noisy background (Salerno et al., 2020). As the result, 3-MeO-PCP was identified in the urine sample from subject A, with a QMF of 90.4 (Hit #1, correct match) vs. a QMF of 18 for the 4-MeO-PCP isomer (Hit #7, incorrect match).

Due to the higher concentration of the target 3-Meo-PCP analyte, only two consecutive depositions were sufficient in the case of a urine sample from subject B, for a QMF increase from 70 (first GC-FTIR analysis) to >90 (second deposition). As for the previous case, a high QMF differential was obtained here too, between the correct (3-MeO-PCP) and the incorrect (4-MEO-PCP) match, viz. 92.9 vs. 12.2.

## Conclusion

The results described in this paper highlight the effectiveness of LC-HRAM-Orbitrap-MS and sd-GC-FTIR in attaining the full structural characterization of 3-MMC and 3-MeO-PCP in seized products, the identification of 3-MeO-PCP and numerous phase I and phase II metabolites in blood, plasma, and urine samples of both cases, and the discrimination between the MMC and MeO-PCP positional isomers [(3-MMC vs. 4-MMC and 2-MMC) and (3-MeO-PCP vs. 4-MeO-PCP)] in both non-biological and biological specimens.

Advanced MS techniques, such as LC-HRAM-Orbitrap-MS, represent valuable analytical tools in forensic toxicology, allowing for highly specific and sensitive untargeted or targeted drug detection in both seized materials and biological fluids. An outstanding analytical specificity is currently attainable; it is allowable through the accurate mass measurement of ionic species, the comparison of experimental and calculated isotopic patterns, and the study of collision-induced product ions obtained in high-resolution conditions. Furthermore, the application of HRMS measurements in full scan conditions avoids the drawbacks of the pre-selection of ions, as in Selected Ion Monitoring (SIM) or Multiple Reaction Monitoring (MRM) acquisition modes. Also, full-scan data may be always retrospectively processed, without resorting to new sample preparation and analytical runs.

Solid-phase GC-FTIR has proven to be an effective tool to widespread MS-based approaches, for achieving unequivocal identification of NPS, even in the case of regioisomers, having identical nominal masses, and yielding the same fragments upon dissociation. Noticeably, confident discrimination between 3-MeO-PCP and related isomers could be achieved from much lower analyte amounts (1 μg powder containing the molecule) compared to NMR approaches, which typically require mg-level quantities (Ameline et al., 2019b). Apart from the seized sample analysis, the application of sd-GC-FTIR to human urine samples demonstrated the usefulness of the technique for the investigation of biosamples of clinical and forensic toxicological concern. Thus, even in the absence of the powder, sd-GC-FTIR analysis of biological samples would have been sufficient to clarify the existence of a specific MeO-PCP positional isomer. Compared to the application of single GC, LC, and MS techniques for the discrimination of MMC and MeO-PCP analogs in non-biological drug samples, which can be problematic due to their quite similar chromatographic and, especially, mass spectrometric, behaviors (Berar et al., 2019), the application of sd-GC-FTIR affords unambiguous identification of positional isomers.

## Data Availability

Due to legal reasons (analytical investigations requested by police for forensic purposes) the raw analytical data supporting the conclusions of this article are not publicly and indiscriminately available. They will be made available by the authors to qualified researchers only, on strictly specific and motivated instances. Any requests to access the raw data should be directed to the corresponding author.
