# Supplementary material for: Analytical Characterization of 3-MeO-PCP and 3-MMC in Seized Products and Biosamples: The Role of LC-HRAM-Orbitrap-MS and Solid Deposition GC-FTIR
Source: Front Chem. 2021 Feb 8;8:618339. doi: 10.3389/fchem.2020.618339 (PMC7897676; doi:10.3389/fchem.2020.618339)
Supplement: Supplementary file 1 [file datasheet1.docx]

**LIST OF CAPTIONS FOR SUPPLEMENTARY FIGURES S1 – S4**

**Figure S1**: 3-MeO-PCP O-demethyl-metabolite (M1a) and 3-MeO-PCP O-demethyl-piperidine-OH-metabolite (M1b) LC-HRAM-Orbitrap-MS full scan mass spectra and MH^+^ collision-induced product ion spectra (collision energies 10, 25, 50 eV) (A), experimental and calculated isotopic patterns of M1a and M1b MH^+^ ions (B), all obtained from LC-HRAM-Orbitrap-MS analysis of urine samples.

**Figure S2**: 3-MeO-PCP O-demethyl-piperidine-di-OH-metabolite (M1g) and 3-MeO-PCP O-demethyl-piperidine-OH-glucuronide-metabolite (M2c) LC-HRAM-Orbitrap-MS full scan mass spectra and MH^+^ collision-induced product ion spectra (collision energies 10, 25, 50 eV) (A), experimental and calculated isotopic patterns of M1g and M2c MH^+^ ions (B), all obtained from LC-HRAM-Orbitrap-MS analysis of urine samples.

**Figure S3**: sd-GC-FTIR analysis of SPE-extracted urine samples from two subjects. GC-FTIR peak at 13.7 min, corresponding to 3-MeO-PCP, with absorption intensities of 0.01 (subject B) and 0.0032 (subject A).

**Figure S4**: Plot of the Quality Match Factor (QMF) value obtained from searching the experimental sd-GC-FTIR spectrum of 3-MeO-PCP in a dedicated library. Multiple deposits of the SPE-extracted urine sample (subject A) afforded an increase in the signal-to-noise ratio and the QMF, accordingly.
