# Supplementary figures and images for: Analytical Characterization of 3-MeO-PCP and 3-MMC in Seized Products and Biosamples: The Role of LC-HRAM-Orbitrap-MS and Solid Deposition GC-FTIR
Source: Front Chem. 2021 Feb 8;8:618339. doi: 10.3389/fchem.2020.618339 (PMC7897676; doi:10.3389/fchem.2020.618339)

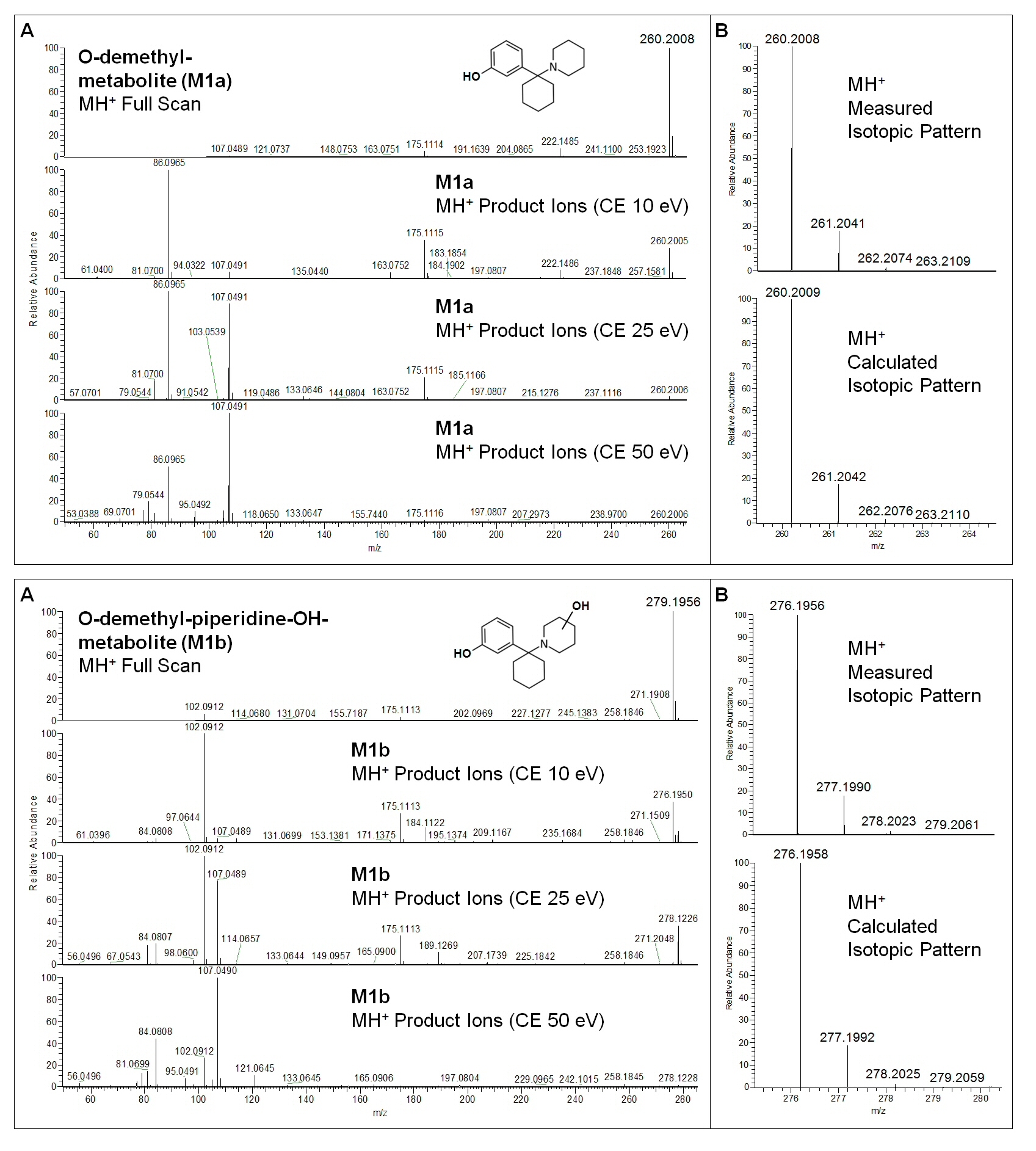

Supplement: Supplementary file 2 [file image1.jpg]

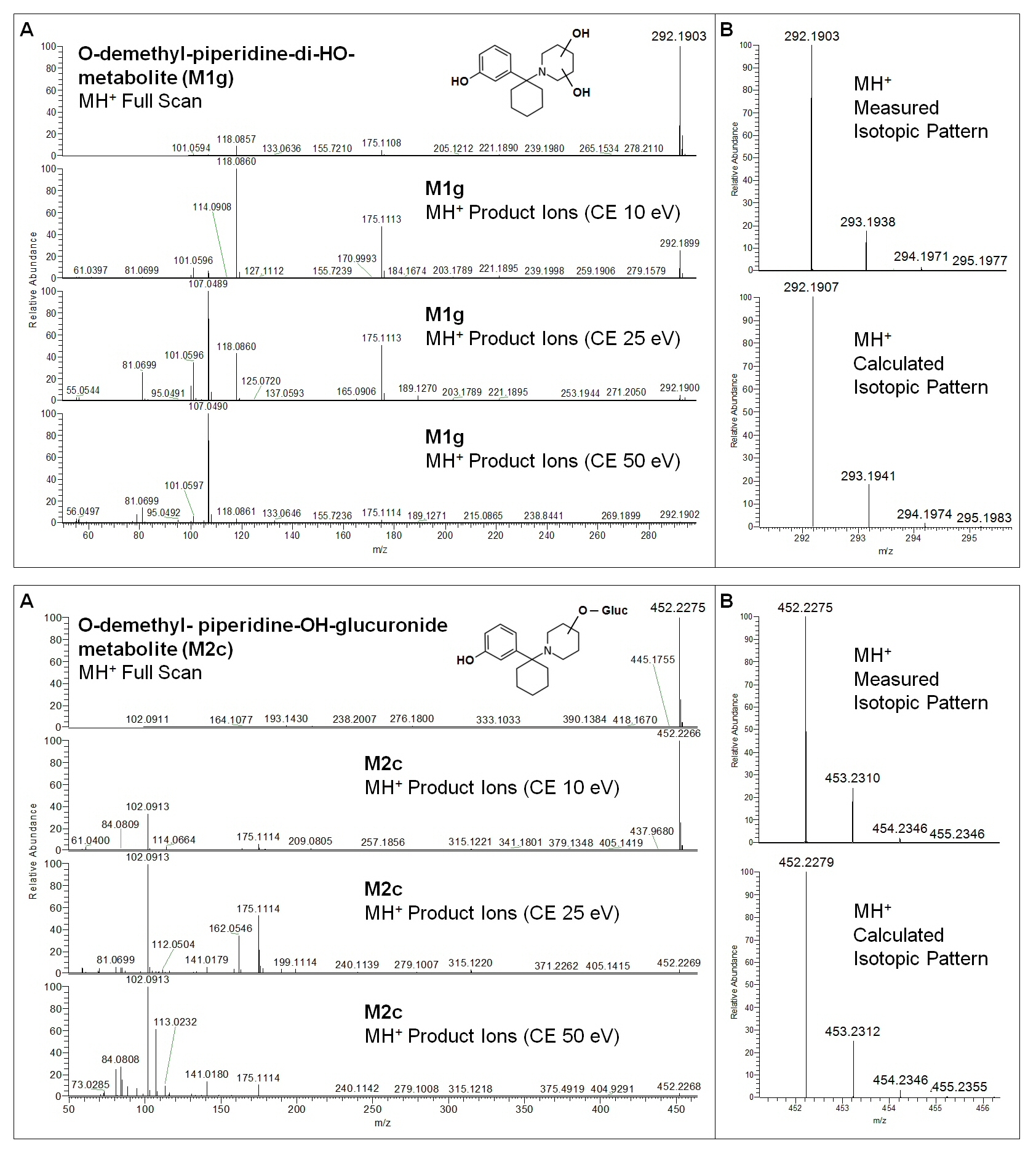

Supplement: Supplementary file 3 [file image2.jpg]

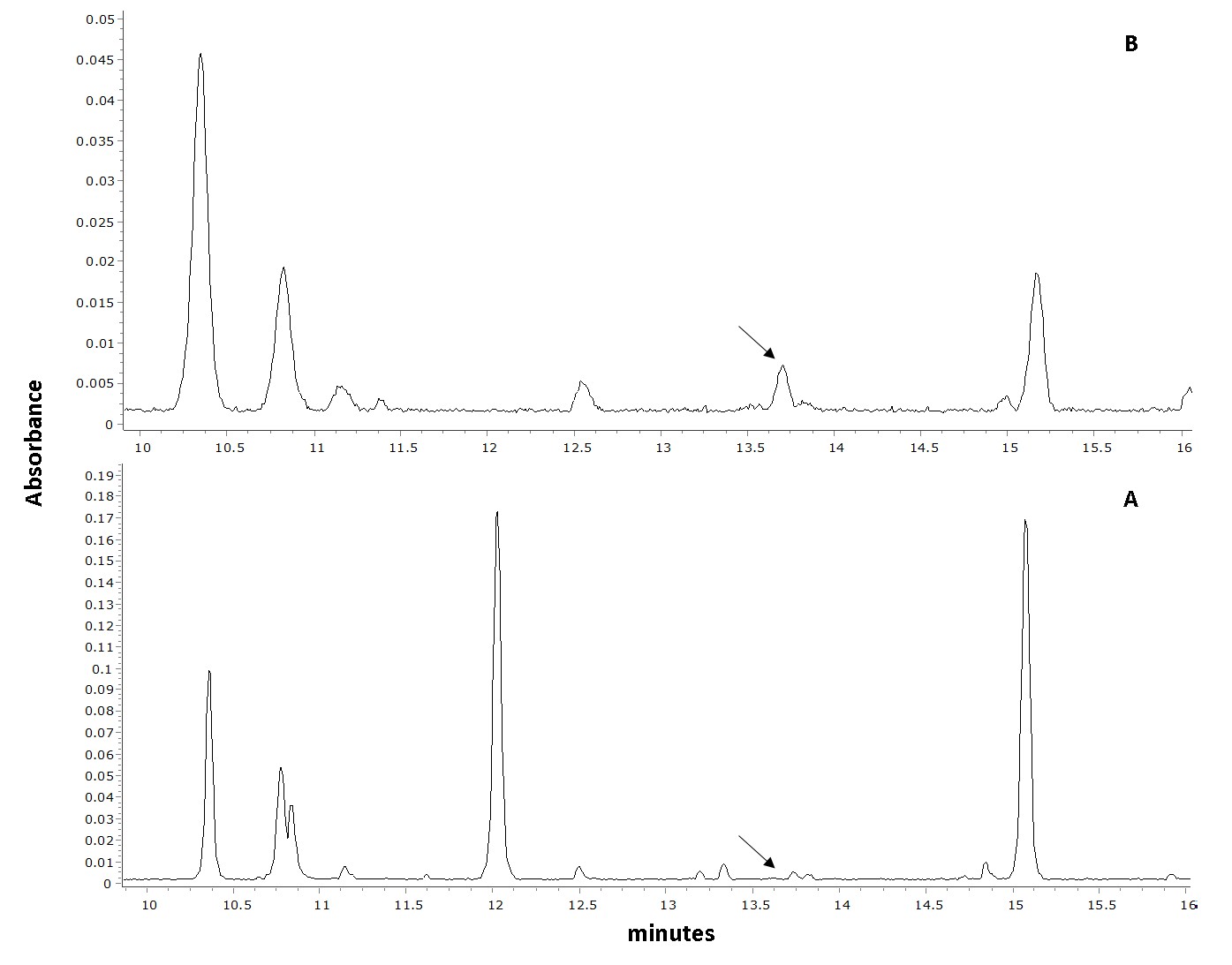

Supplement: Supplementary file 4 [file image3.jpg]

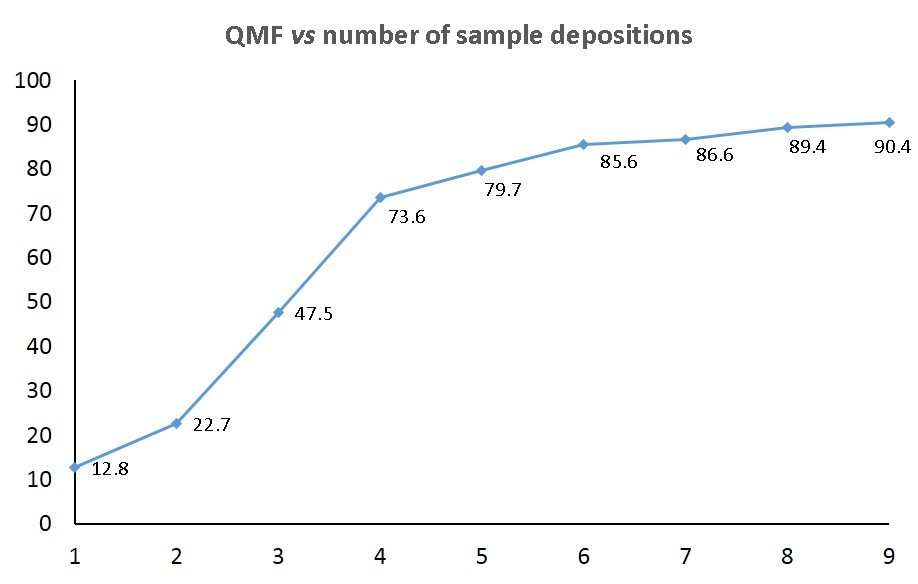

Supplement: Supplementary file 5 [file image4.jpg]
